# How we approach titrating PEEP in patients with acute hypoxemic failure

**DOI:** 10.1186/s13054-023-04694-1

**Published:** 2023-10-31

**Authors:** Leo Heunks, Lise Piquilloud, Alexandre Demoule

**Affiliations:** 1 Department of Intensive Care Medicine, Erasmus Medical Center, Dr Molewaterplein 40, 3015GD Rotterdam, The Netherlands; 2 Department of Intensive Care, Radboudumc, Nijmegen, The Netherlands; 3 Adult Intensive Care Unit, University Hospital and University of Lausanne, Lausanne, Switzerland; 4 INSERM, UMRS1158 Neurophysiologie Respiratoire Expérimentale Et Clinique, Sorbonne Université, Paris, France; 5 Service de Médecine Intensive - Réanimation (Département R3S), AP-HP, Groupe Hospitalier Universitaire APHP-Sorbonne Université, Site Pitié-Salpêtrière, Paris, France

## Introduction

In the recent ESICM guidelines on Acute Respiratory Distress Syndrome [1], the group of experts was “unable to make a recommendation for or against routine PEEP titration with higher PEEP/FiO_2_ strategy versus a lower PEEP/FiO_2_ strategy.” Also, the committee was “unable to make a recommendation for or against PEEP titration guided by respiratory mechanics, compared to PEEP titration principally on PEEP/FiO_2_ strategy.” These recommendations are undoubtedly sound from a methodological perspective but leave the clinicians at the bedside in dire straits. At the bedside, we need to decide regarding the best PEEP for a specific patient at a specific point in time. In this short comment, we share our approach on titrating PEEP at the bedside in patients with acute respiratory distress syndrome (ARDS).

### What are we aiming for with PEEP?

In the original ARDS description by Ashbaugh [2], it was already reported that applying PEEP improves oxygenation (*N* = 5). For a long time, the primary reason to apply PEEP was to improve arterial oxygenation. However, when it became clear that inhomogeneous lung tissue may play an important role in the pathophysiology of VILI, the major aim for PEEP titration was to get a more homogeneous lung tissue or, in other terms, to find an optimal balance between lung recruitment and overdistension. Also, clinicians should be aware of the detrimental effects of (high) PEEP on hemodynamics, especially right ventricular function. Therefore, at the bedside we need to find the right balance between lung recruitment and hyperinflation, while closely monitoring the hemodynamic response.

### What do the RCTs tell us?

**Fig. 1:**
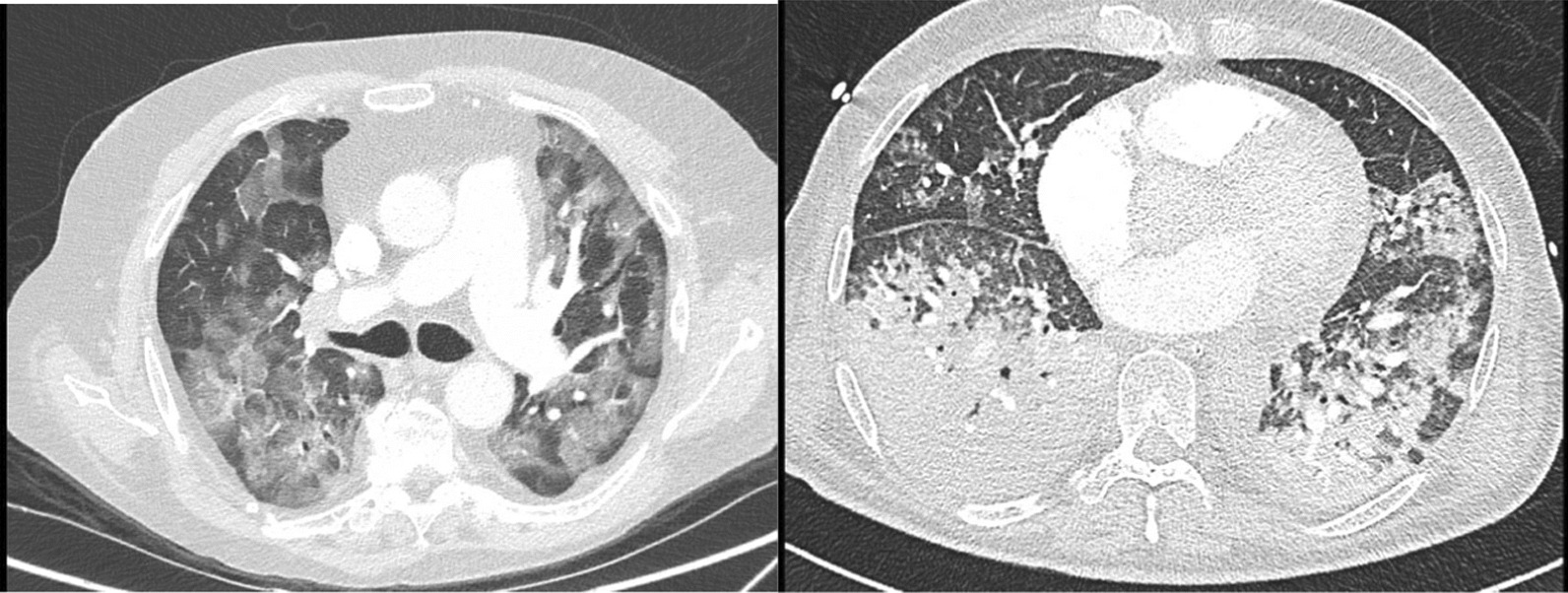
CT scan of two ARDS patients with different potential for lung recruitment. This figure shows images of two different patients, both fulfilling the criteria for ARDS. The CT scan in the left panel is characterized by extensive ground glass appearances, but limited consolidations, whereas the patient in the right panel has extensive gravity dependent consolidations. Obviously, the potential for lung recruitment with PEEP is much higher in the patients in the right panel

Today, three large RCTs have from slightly different perspective compared the effect of lower versus higher PEEP on clinical outcome. These studies are summarized in Table 1. These studies did not show a clear benefit from one approach versus the other. It is important to understand why these trails did not demonstrate an effect of one PEEP approach against the other. This can be understood if we look at the two CT scans in Fig. 1. Both patients present with acute hypoxemic failure, requiring endotracheal intubation. The CT scan in the left panel shows limited consolidations, while consolidations are extensive in the CT scan at the right panel. It is easy to understand that higher PEEP may be beneficial in the patient in the right panel (potentially recruitable lung), while it will only facilitate pulmonary hyperinflation in the other patient. Recently, a multicenter trial showed that aggressive lung recruitment maneuvers associated with PEEP titration according to respiratory system compliance increased mortality of patients with moderate to severe ARDS [3]. Application of high PEEP levels in patients with little potential for lung recruitment may have contributed to poor outcomes in this study. Thus, the discussion whether PEEP in patients with acute hypoxemic failure should be set “low” or “high” does not make much sense from a physiological perspective.

**Table 1:** Summary of the three clinical trials evaluating “lower or higher PEEP” setting

Trial	N	Patients	Lower PEEP	Higher PEEP	Primary outcome
Alveoli [5]	549*	Early ARDS, Pao_2_/FiO_2_ < 300mmHg, inclusion within 36 h of meeting eligibility criteria	Low PEEP/Fi,O_2_ table	High PEEP/Fi,O_2_ table	Proportion of patients who died before they were discharged home while breathing without assistance was not different between groups (25% vs 28%)
LOV [13]	983	Early ARDS, Pa,O_2_/Fi,O_2_ < 250mmHg, inclusion within 48 h of meeting eligibility criteria	Lower PEEP/Fio_2_ table	High PEEP/Fi,O_2_ table. Protocol modifications during trial	All-cause hospital mortality was not different between groups (40% vs 35%)
Express [8]	767	Early ARDS, Pa,O_2_/Fi,O_2_ < 300mmHg, inclusion within 48 h of meeting eligibility criteria	PEEP between 5 and 9 cmH_2_O	As high as possible while maintaining Pplat < 30cmH_2_O	28-day motility was not different between groups (31% vs 28%)

*Estimated sample size was 750 patients, but this trial was stopped after 549 patients on the basis of the specified futility stopping rule

A higher PEEP may only be beneficial in patients with potential for lung recruitment, but none of the three RCT’s evaluated the potential for lung recruitment prior to PEEP selection. Although not specifically evaluating higher versus lower PEEP, Constantin and colleagues [4] compared personalized mechanical ventilation (based on lung morphology assessed by chest X-ray or chest CT scan) versus standard of care. In the control group, PEEP was set according to low PEEP/ FiO_2_ table and TV was set at 6ml/Kg predicted bodyweight (PBW). In the personalized group, patients with focal ARDS received TV of 8ml/Kg PBW and PEEP between 5 and 9cmH_2_O. In the non-focal ARDS group, TV was set at 6ml/Kg PBW and PEEP titrated to reach an inspiratory plateau pressure of 30cmH_2_O. Of note, recruitability with PEEP was not evaluated. No significant difference was found in 90-day mortality between control group or personalized ventilation group. However, it appeared that 21% of patients in the personalized group were misclassified (focal versus non-focal ARDS) and that misclassified patients had *higher* mortality as comparted to control group and to correctly classified patients. This study could be interpreted that a personalized approach to ventilator settings may have a beneficial effect on clinical outcome. Though, this remains to be established.

### ***A practical approach (***Fig. 2***)***

**Fig. 2:**
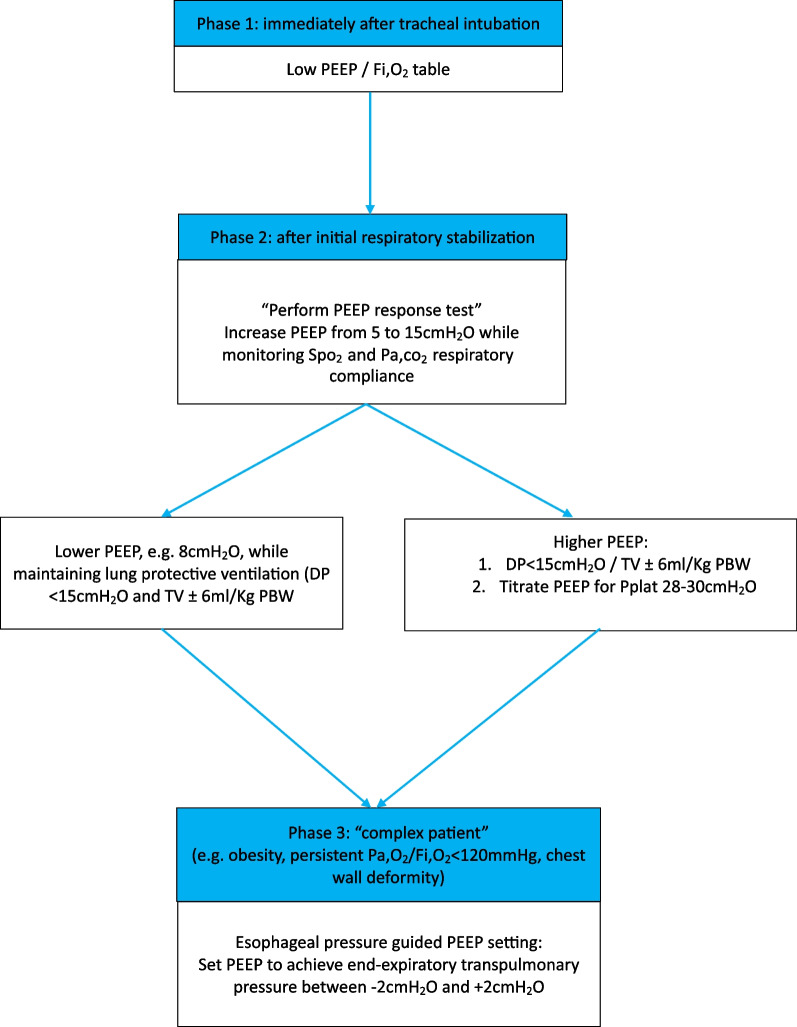
Practical algorithm for PEEP titration in patients with acute hypoxemic failure

In patients with moderate and severe acute hypoxemic failure, we set PEEP immediately after endotracheal intubation according to the *low* PEEP/FiO_2_ table [5], which means in clinical practice a PEEP between 8 and 12cmH_2_O (with Fio_2_ between 0.5 and 0.7). Of course, in patients with low chest wall compliance (e.g. obesity, ascites), higher PEEP levels may be selected. Although selecting the *high* PEEP/Fio2 table may seem justified by a meta-analysis demonstrating that higher PEEP/FiO_2_ may present benefit on clinical outcome in these patients [6], none of these studies evaluated the potential for lung recruitment. Moreover, it seems that in clinical practice, few clinicians are willing to select PEEP based on high PEEP/Fi,o_2_ table [7].

Shortly after endotracheal intubation, we identify whether the patient we are managing has potential for lung recruitment. To this aim, we perform a PEEP-responsiveness test, which consists in increasing PEEP from 5 to 15cmH_2_O in a single step. Response is evaluated after 10 min. If compliance and oxygenation do not decrease (arbitrarily 10% and 2% of Spo_2_, respectively) and if PaCO_2_ does not increase (arbitrarily 10%), this patient has probably potential for lung recruitment and hence may benefit from higher PEEP level. We thus increase PEEP level (with maintaining TV ± 6ml/Kg PBW and driving pressure < 15cmH_2_O) until respiratory system static plateau pressure reaches 28-30cmH_2_O, according to the ExPress trial approach [8]. If the patient does not have potential for lung recruitment, we set a moderate PEEP level, e.g., 8cmH_2_O. Potential for lung recruitment is evaluated daily and after transition to prone position (vice versa).

However, in some patients, identifying the optimal PEEP may be more challenging, for instance, in patients with obesity because they could have low chest wall compliance, increased intraabdominal pressure, or very severe hypoxemic failure. In such patients, we use advanced respiratory monitoring to evaluate the impact of PEEP at the bedside. Measurement of esophageal pressure provides an estimation of global pleural pressure that allows calculating global transpulmonary pressure. Physiologically, it may be attractive to titrate PEEP to obtain end-expiratory transpulmonary pressure around zero, limiting alveolar collapse. This approach has been evaluated in the Epvent-2 trial, although PEEP was adjusted to achieve an end-expiratory transpulmonary pressure between 0 and 6 cmH_2_O. In the control group, PEEP was titrated using the high PEEP/FiO_2_ table. The primary outcome, a composition score incorporating death and ventilator free days through day 28, was not different between the two groups. In a reanalysis of this study [9], it was demonstrated that titrating PEEP to transpulmonary end-expiratory pressure close to zero (± 2cmH_2_O) was associated with greater survival as compared to more positive or negative values. It should, however, be noted that application of esophageal pressure measurement as an estimation of pleural pressure may be complex and requires expertise. Practicalities for esophageal pressure manometry have been extensively discussed previously [10].

## PEEP and the right ventricle

PEEP may increase pulmonary vascular resistance and such right ventricle afterload further deteriorating right ventricle function [11]. In patients with high driving pressure (≥ 18 cmH_2_O), severe hypoxemia or hypercapnia (≥ 48 cmH_2_O), routine echocardiography should be performed to search for acute cor pulmonale [12]. If cor pulmonale develops or deteriorates with higher PEEP, it may be advisable to decrease PEEP and prioritize hemodynamics. Respiratory rate may also be increased in parallel in the absence of dynamic airtrapping, to the aim of reducing PaCO_2_, which contributes to pulmonary hypertension.

### Other techniques to quantify PEEP-induced lung recruitment

Other techniques have been used to quantify lung recruitment by PEEP, including CT scan, stress index, ultrasound, electrical impedance tomography (EIT) and recruitment-to-inflation ratio (assessment of de-recruitment with decreasing PEEP). However, these techniques are not feasible bedside (CT scan) or require further validation in clinical practice (other techniques).

### Conclusion

The decision to titrate PEEP at the bedside may be complex and without clear guidelines. In this manuscript, we outline an approach based on physiology and clinical experience.

## Data Availability

Not applicable.
